# Developmental programming: rescuing disruptions in preovulatory follicle growth and steroidogenesis from prenatal testosterone disruption

**DOI:** 10.1186/s13048-016-0250-y

**Published:** 2016-06-29

**Authors:** A Veiga-Lopez, J Moeller, D. H. Abbott, V Padmanabhan

**Affiliations:** Department of Pediatrics, University of Michigan, 7641A Med Sci II, Ann Arbor, MI 48109-5622 USA; Department of Animal Science, Michigan State University, East Lansing, MI 48824 USA; Department of Obstetrics and Gynecology and Wisconsin National Primate Research Center, University of Wisconsin, Madison, WI 53715 USA

**Keywords:** Steroids, Testosterone, Androgen antagonist, Insulin sensitizer

## Abstract

**Background:**

Prenatal testosterone (T) excess from days 30-90 of gestation disrupts gonadotropin surge and ovarian follicular dynamics and induces insulin resistance and functional hyperandrogenism in sheep. T treatment from days 60-90 of gestation produces a milder phenotype, albeit with reduced fecundity. Using this milder phenotype, the aim of this study was to understand the relative postnatal contributions of androgen and insulin in mediating the prenatal T induced disruptions in ovarian follicular dynamics.

**Methods:**

Four experimental groups were generated: 1) control (vehicle treatment), 2) prenatal T-treated (100 mg i.m. administration of T propionate twice weekly from days 60-90 of gestation), 3) prenatal T plus postnatal anti-androgen treated (daily oral dose of 15 mg/kg/day of flutamide beginning at 8 weeks of age) and 4) prenatal T and postnatal insulin sensitizer-treated (daily oral dose of 8 mg/day rosiglitazone beginning at 8 weeks of age). Follicular response to a controlled ovarian stimulation protocol was tested during their third breeding season. Main outcome measures included the determination of number and size of ovarian follicles and intrafollicular concentrations of steroids.

**Results:**

At the end of the controlled ovarian stimulation, the number of follicles approaching ovulatory size (≥6 mm) were ~35 % lower in prenatal T-treated (6.5 ± 1.8) compared to controls (9.8 ± 2.0). Postnatal anti-androgen (10.3 ± 1.9), but not insulin sensitizer (5.0 ± 0.9), treatment prevented this decrease. Preovulatory sized follicles in the T group had lower intrafollicular T, androstenedione, and progesterone compared to that of the control group. Intrafollicular steroid disruption was partially reversed solely by postnatal insulin sensitizer treatment.

**Conclusions:**

These results demonstrate that the final preovulatory follicular growth and intrafollicular steroid milieu is impaired in prenatal T-treated females. The findings are consistent with the lower fertility rate reported earlier in these females. The finding that final follicle growth was fully rescued by postnatal anti-androgen treatment and intrafollicular steroid milieu partially by insulin sensitizer treatment suggest that both androgenic and insulin pathway disruptions contribute to the compromised follicular phenotype of prenatal T-treated females.

## Background

With well over 5 million U.S. women affected, women with polycystic ovary syndrome (PCOS) are frequent patients in infertility clinics, seeking assistance in becoming pregnant [1]. A PCOS diagnosis is reached when two of the following three criteria are met: hyperandrogenism, oligo- or anovulation, and/or polycystic ovaries [2–4]. The reduced pregnancy rate in PCOS patients has been attributed to the oligo-anovulatory condition of the syndrome; this, in part, stems from a disrupted intrafollicular milieu, which includes reductions in cortisone [5], insulin growth like factor (IGF) I and II [6], and progesterone (P4) [7], increases in anti-Mullerian hormone (AMH) [8], testosterone, androstenedione, and proteomic dysregulation [9]. The compromised intrafollicular steroidal milieu in PCOS women likely accounts for the poor quality of oocytes [10, 11]. A recent meta-analysis study found removal of oocytes from the disrupted endogenous steroidal environment of PCOS women and maturing them in vitro helps achieve better conception rates [12]. Understanding the dysregulation of the intrafollicular milieu is essential for developing strategies to overcome infertility in PCOS.

Increasing evidence from several species (rhesus monkeys, sheep, rats, and mice) has demonstrated a link between prenatal exposure to testosterone (T) and development of a PCOS-like phenotype [13, 14]. Specifically, prenatal T-treatment disrupts the intrafollicular steroidal balance in preovulatory follicles (5-7 mm) and reduces embryonic potential in rhesus monkeys [11]. In sheep, prenatal T excess from days 30 to 90 of gestation (T30-90) enhances follicular recruitment and persistence [13] and causes disruptions in several key mediators of folliculogenesis [15–18]. A milder PCOS-like phenotype with reduced fecundity was found in sheep treated prenatally from days 60-90 of gestation (T60-90), where only 40 % of such prenatal T-treated females became pregnant [19]. These T60-90 females also developed insulin resistance [13].

Using the milder T60-90 phenotype [19] and a controlled ovarian stimulation protocol that effectively stimulates follicular development [20], we tested the hypothesis that prenatal T excess compromises maturation of the preovulatory follicle and disrupts the intrafollicular milieu in sheep. Since *i*) prenatal T-treated sheep manifest functional hyperandrogenism and insulin resistance [13]; *ii*) treatment with an androgen antagonist or insulin sensitizer improves ovulatory function in women with PCOS [21], the reproductive phenotype of whom prenatal T-treated sheep recapitulate; and *iii*) postnatal insulin sensitizer-treatment prevents a progressive loss in cyclicity of prenatal T-treated sheep [22], this study aimed to parse out the relative postnatal contribution of androgen and insulin towards dysfunctional follicle responses of T60-90 prenatal T-treated sheep to controlled ovarian stimulation in adulthood.

## Methods

### Prenatal and postnatal treatments

All procedures used were approved by the Institutional Animal Care and Use Committee of the University of Michigan and conducted at the University of Michigan Sheep Research Facility. Animal husbandry details have been published previously [23]. Mature Suffolk ewes (2 to 3 years in age) maintained under a natural photoperiod were mated and date of mating confirmed based on rump paint marks left by a raddled ram. Pregnant ewes were blocked by weight and body score and randomly assigned to one of two treatment groups: 36 animals in the prenatal T treatment group received 100 mg T propionate (Sigma-Aldrich Corp., St. Louis, MO) twice weekly in 2 ml of corn oil, i.m. from days 60 to 90 of gestation, while 12 controls received an equal volume of vehicle. Before puberty beginning at 8 weeks of age, prenatal T-treated females received either androgen antagonist, flutamide (Sigma-Aldrich, Corp.) (*n* = 11), the insulin sensitizer, rosiglitazone (Avandia; GlaxoSmithKline, Durham, NC) (*n* = 12), or no treatment (*n* = 13). Flutamide was administered orally at a dose of 15 mg/kg/ewe/day and rosiglitazone orally at a dose of 0.11 mg/kg/ewe/day as previously described [24].

### Controlled ovarian stimulation

**Fig. 1:**
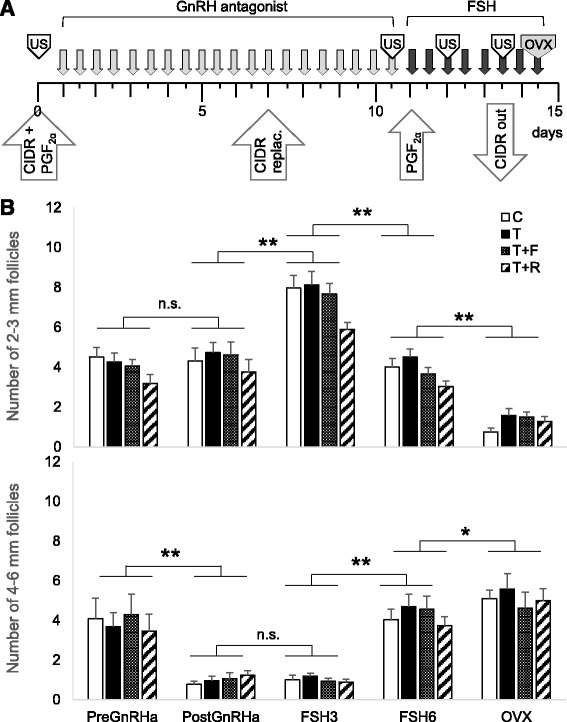
**a** Scheme depicting synchronization and controlled ovarian stimulation protocol used in the study. See text for details. CIDR: intravaginal P4 control internal drug release device, PGF_2α_: prostaglandin F_2α_, FSH: follicle stimulating hormone, GnRH: gonadotropin releasing hormone, OVX: ovariectomy, replac.: replacement, US: transrectal ultrasonography. Grey and black arrows indicate time of GnRH antagonist and FSH administration, respectively. **b** Mean (± SEM) number of 2-3 mm (*top panel*) and 4-6 mm (*bottom panel*) before GnRH antagonist (PreGnRHa), after GnRH antagonist (PostGnRHa), before the 3^rd^ and 6^th^ FSH doses (FSH3 and FSH6, respectively), and ovariectomy (OVX) in control (*white bars*), T (*filled bars*), T + F (*dotted bars*), and T + R (*stripped dars*) females. See Fig. 1 for synchronization and controlled ovarian stimulation protocol and text for details of prenatal/postnatal treatment details. F: flutamide; R: rosiglitazone; n.s.: not significant; * *P* < 0.01, ** *P* < 0.001

During their third breeding season (~2.5 years of age), the follicular response to a controlled ovarian stimulation protocol modified from that previously described [25] was tested in all females (see Fig. 1a). All females received 2 ml of prostaglandin F_2α_ (PGF_2α_, 5 mg/ml; Lutalyse, Pfizer Animal Health, MI) and an intravaginal P4 control internal drug release device (CIDR; Eazi-Breed CIDR sheep inserts, Pfizer Animal Health, NY) on day 0 that was replaced on day 7. Beginning on day 1, 10 μg/kg body weight of acyline, a GnRH antagonist (GnRHa) procured from the National Hormone and Peptide Program, was administered every 12 h for 10 days. This was followed by administration of 8 decreasing doses (two doses at each concentration) of FSH (0.6, 0.4, 0.3, and 0.1 mg/kg; Folltropin-V, Bioniche Animal Health, GA) starting on day 11. The P4 CIDR was removed after the sixth FSH dose.

### Follicular dynamics

To monitor changes in follicular dynamics, transrectal ultrasonography was performed as previously described [26] using a scanner (Aloka SSD-900 V, Aloka Co. Ltd., Wallington, CT) fitted to a 7.5 MHz linear array transducer. Number of follicles ≥ 2 mm and corpora lutea were determined prior to the start of GnRHa treatment, after the last GnRHa dose, and after the 3^rd^ and 6^th^ FSH dose (Fig. 1a). Two hours after the 8^th^ FSH dose, a subset (n = 6/group) of females were ovariectomized following procedures previously described [27], and all follicles ≥ 3 mm were dissected [20]. After recording their diameter, follicular fluid was aspirated and frozen at -20 °C. Prior to measurements, all follicular fluids were diluted 1:100 in 1x PBS supplemented with 1 % BSA. After follicular dissection and aspiration, the collapsed follicle was not useful to undertake histological studies.

### Intrafollicular steroids

Follicular fluid concentrations of androstenedione (A4), estradiol (E_2_), estrone, P4, and T were measured by quadruple linear ion trap mass spectrometer (LC-MS/MS) from one 3 mm, one 4 mm, and two 5-6 mm follicles that were randomly selected from each ovariectomized female. Samples (400 μl) were extracted after diluting with ultrapure water (500 μl). An internal deuterated standard and 1 ml of 2-methoxy-2-methylpropane was added to each sample, vortexed vigorously, and incubated for 5 min at room temperature. The steroid-containing organic phase was air-dried and re-suspended in 100 μl of ethanol and 500 μl of water. A second liquid-liquid extraction was performed with dichloromethane. The steroid-containing dichloromethane phase was air-dried and samples re-suspended in NaHCO_3_ buffer (25 μl), and estrone and E_2_ were derivitized with 50 μl of dansyl chloride (200 mg/ml in acetonitrile), heated to 40 °C for 4 min, and transferred into minivials.

A4, T, E_2_, estrone, and P4 were assayed in the Assay Services Laboratories at the Wisconsin National Primate Research Center using a QTRAP 5500 LC-MS/MS (AB Sciex, USA) equipped with an atmospheric pressure chemical ionization source. The system included two Shimadzu LC20ADXR pumps and a Shimadzu SIL20ACXR autosampler. Thirty μl samples were injected onto a Phenomenex Kinetex 2.6u C18 100A, 100 × 2.1 mm column (Phenomenex) for separation. LC-MS/MS results were generated in positive-ion mode with optimized voltages. Calibration curve concentrations for estrogens were 1.56-0.003 ng/ml and 3.91-0.0076 ng/ml ng/ml for remaining steroids. Linearity was r > 0.9990 and curve fit was linear with 1/x weighting. Interassay coefficients of variation were determined by a pool of human serum and ranged from 6.09-19.47 % for all steroids. Assay sensitivities for A4, E_2_, estrone, P4 and T were 0.015, 0.005, 0.0015, 0.015, and 0.0325 ng/ml, respectively.

### Statistical analysis

For analyses of follicular dynamics, follicles were grouped as 2-3 mm, 4-6 mm, ≥2 mm, ≥4 mm, and ≥6 mm follicles. For intrafollicular steroid measurements, follicle classes included 3 mm, 4 mm, and 5-6 mm in diameter. Follicle size distribution among treatment groups and intrafollicular steroid concentrations among follicular classes within each treatment group and within a follicular class across treatment groups were analyzed by ANOVA and linear mixed effect model with Tukey posthoc tests. Percent change in intrafollicular steroid concentrations between 3 mm and 5-6 mm follicles was derived by subtracting concentration in 3 mm from that in larger follicles. Appropriate transformations were applied, as needed, to account for normality of data allowing analyses by parametric tests. All analyses were carried out using PASW Statistics for Windows release 18.0.1 and data presented as mean ± SEM. *P* < 0.05 was considered significant.

## Results

### Follicular size dynamics

GnRHa treatment decreased the number of 4-6 mm, but not 2-3 mm, follicles (*P* < 0.001; Fig. 1b). FSH administration increased number of 2-3 mm follicles (*P* < 0.001) by the third dose followed by a decline by the 6^th^ FSH dose (*P* < 0.001). This decline in 2-3 mm was accompanied by a marked increase (*P* < 0.001) in the number of 4-6 mm follicles (*P* < 0.001). At ovariectomy, a further decline in 2-3 mm follicles and an increase in 4-6 mm follicles (P < 0.001) were found. GnRHa- and FSH-induced changes in 2-3 and 4-6 mm follicles in all treatment groups did not differ from the control group.

**Fig. 2:**
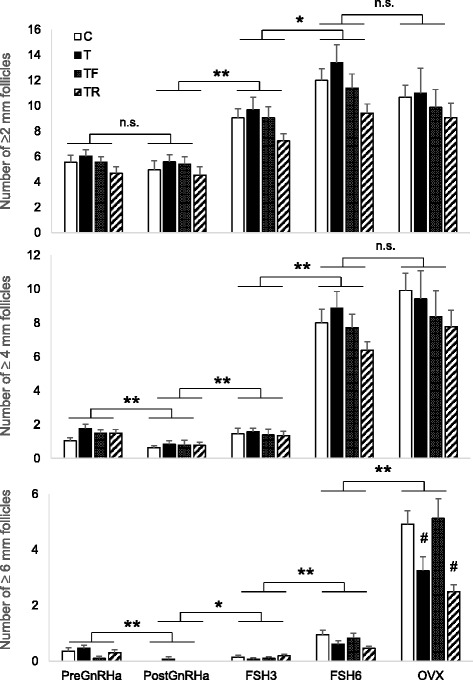
Mean (± SEM) number of ≥2 mm (*top panel*), ≥ 4 mm (*middle panel*), and ≥ 6 mm (*bottom panel*) follicles before GnRH antagonist (PreGnRHa), after GnRH antagonist (PostGnRHa), before the 3^rd^ and 6^th^ FSH dose (FSH3 and FSH6, respectively), and ovariectomy (OVX) in control (C; *white bars*), T (*filled bars*), T + F (*dotted bars*), and T + R (*stripped bars*) females. See Fig. 1 for synchronization and controlled ovarian stimulation protocol and text for prenatal/postnatal treatment details. F: flutamide; R: rosiglitazone; n.s.: not significant; * *P* < 0.01, ** *P* < 0.001. # represents significant different (*P* < 0.05) compared to the control group

When all follicles ≥2 mm were considered, FSH increased the total number of follicles by the third FSH dose (*P* < 0.001; Fig. 2), increasing further until the sixth FSH dose (*P* < 0.001) but not beyond. In contrast, GnRHa administration significantly reduced ≥4 mm follicles (*P* < 0.001; Fig. 2). An increase in ≥4 mm follicles was observed following the third FSH dose and beyond (*P* < 0.001). There were no differences in follicular classes ≥2 mm and ≥4 mm between control and all treatment groups.

An increase was evident by the sixth FSH dose in ≥6 mm follicles (*P* < 0.001), culminating in a 3-fold increase at ovariectomy (*P* < 0.001; Fig. 2). Prenatal T treatment reduced ≥6 mm follicle number, with an initial decline evident by sixth FSH dose and achieving significance at ovariectomy (Fig. 2). Postnatal treatment with flutamide, but not rosiglitazone, prevented the prenatal T-induced reduction in ≥6 mm follicles.

### Intrafollicular steroids

**Fig. 3:**
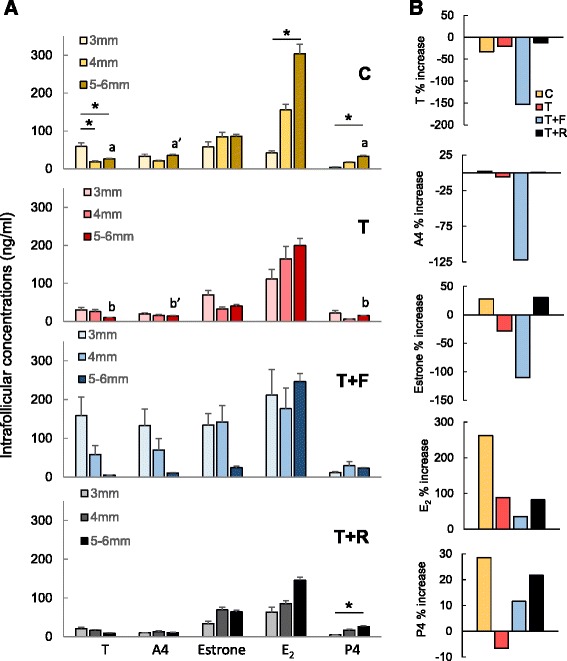
**a** Mean (± SEM) intrafollicular concentrations (ng/ml) of testosterone (T), androstenedione (A4), estrone, estradiol (E_2_), and progesterone (P4) at the time of ovariectomy and coincidentally with the 8^th^ FSH dose in control (C; *yellow*), prenatal testosterone-treated (T; *red*), prenatal T plus postnatal flutamide (T + F; *blue*), and prenatal T plus postnatal rosiglitazone (T + R; *gray*) females. See Fig. 1 for synchronization and controlled ovarian stimulation protocol and text for prenatal/postnatal treatment details. Within group comparisons: asterisks represent differences within group within hormone and between follicular sizes. Posthoc analyses performed only when overall ANOVA among all three sizes was significant. Comparisons between C and T group are represented by a ≠ b if P < 0.05 and by a’ ≠ b’if P = 0.07. F: flutamide; R: rosiglitazone. Data obtained from one 3 mm, one 4 mm, and two 5-6 mm follicles randomly selected from each female. **b** Percent change in intrafollicular steroids of T, A4, estrone, E_2_, and P4 between 3 and 5-6 mm follicles in C (*yellow*), T (*red*), T + F (*blue*), T + R (*black*) groups. Percent change was calculated by subtracting the overall mean values between the 3 and 5-6 mm size within each group

Figure 3a shows changes in intrafollicular concentrations of steroids. In control females, intrafollicular T concentrations were higher in 3 mm vs. larger follicles (*P* < 0.05), while the reverse was found for intrafollicular E_2_ and P4, with higher concentrations found in 5-6 mm follicles (*P* < 0.05). Control females had an increase in E_2_ with follicle size (*P* < 0.05) that was not seen in T and T + R females. T + F females had high E_2_ concentrations regardless of follicle size. There was no follicle size effect on intrafollicular T and E_2_ concentrations in T and T + F females, while an increase in P4 was evident in 5-6 mm follicles of T + R females.

No differences were found among treatment groups with intrafollicular T, A4, estrone, E_2_, and P4 concentrations in the different follicular classes. When analysis was restricted only to control and T-treated females, T females had lower T and P4 (*P* < 0.05) and tended to have lower A4 (*P* = 0.07) in 5-6 mm follicles. Overall evaluation of change in steroids between the 3 and 5-6 mm sized follicles (Fig. 3b) revealed increases in A4, estrone, E_2_, and P4 and a reduction in T in control females, while reductions in A4, estrone, and P4 were observed in T females. The increase in E_2_ from 3 to 5-6 mm was of a higher magnitude in control compared to T females. The changes in A4, estrone, and E_2_ were more pronounced in T + F than T females. The directionality of changes in steroids between 3 and 5-6 mm in T + R females mirrored that of control females.

**Fig. 4:**
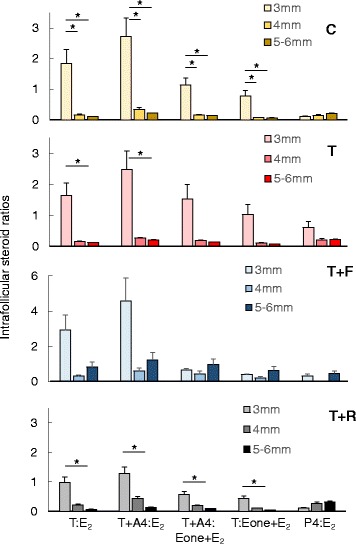
Mean (± SEM) intrafollicular steroid ratios of testosterone to estradiol (T: E_2_), T plus androstenedione (A4) to E_2_ (T + A4:E_2_), T + A4:estrone + E_2_, T:estrone + E_2_, and P4:E_2_ in 3 mm, 4 mm, and 5-6 mm follicles at the time of ovariectomy and coincidentally with the 8^th^ FSH dose in control (C; *yellow*), prenatal testosterone-treated (T; *red*), prenatal T plus postnatal flutamide (T + F; *blue*), and prenatal T plus postnatal rosiglitazone (T + R; *gray*) females. See Fig. 1 for synchronization and controlled ovarian stimulation protocol and text for prenatal/postnatal treatment details. Within group comparisons, asterisks represent differences within group within hormone and between follicular sizes. Posthoc analyses performed only when overall ANOVA among all three sizes was significant. Data obtained from one 3 mm, one 4 mm, and two 5-6 mm follicles randomly selected from each female

Comparison of steroid ratios across follicular stages found the follicular androgen to estrogen ratios (T:E_2,_ T + A4:E_2,_ T + A4:estrone + E_2,_ and T:estrone + E_2_) in controls were lower in 5-6 mm compared 3 mm follicles (*P* < 0.05) (Fig. 4). The T:E_2_ and T + A4:E_2_ ratios were also lower in 5-6 mm vs. 3 mm follicles in T females. Intrafollicular T:E_2_, T + A4:E_2_, T + A4:estrone + E_2_, and T:estrone + E_2_ ratios were all lower (*P* < 0.05) in 5-6 mm vs. 3 mm follicles within the T + R group. No treatment effect was found in steroid ratios. Data skewness prevented detection of differences in T:E_2_ and T + A4:E_2_ ratios in T + F females.

## Discussion

This study is the first to demonstrate that prenatal T excess from days 60-90 of gestation impairs final preovulatory follicular growth (ovulatory size: ≥6 mm; [28]) and reduces intrafollicular concentrations of T, A4, and P4 in preovulatory sized follicles of sheep undergoing controlled ovarian stimulation, a finding consistent with the lower fertility rates reported in these sheep [19]. The rescue of final growth by postnatal androgen antagonist, but not insulin sensitizer, administration suggests the impairment of prevoulatory follicle growth is mediated via androgenic action. This, in concert with the partial rescue of the intrafollicular steroid milieu with insulin sensitizer, suggests that both androgens and insulin contribute to reproductive disruption [29–31] and reduced fecundity [17] in adult prenatal T-treated sheep.

### Prenatal T programming of follicular dynamics

The GnRHa treatment regimen used was effective in blocking follicular growth beyond the 3 mm stage in both the control and prenatal T-treated sheep, as was the case with T30-90 females exposed to a shorter GnRHa treatment [32]. The efficacy of pFSH to recruit follicular growth in controls was comparable to that achieved with oFSH or pFSH stimulation in other sheep breeds [25, 33]. pFSH used in this study was also as effective in stimulating follicular growth in the 3 treatment groups (T, T + F, and T + R) as the combined oFSH- LH regimen used with T30-90 females [32]. These findings indicate responsiveness to exogenous FSH, and hence recruitment, was not impaired by prenatal T excess or postnatal treatment with androgen antagonist or insulin sensitizer. The finding that recruitment and growth of follicles up to 4 mm size was similar across groups is not surprising, because androgens increase FSH activity [34] and are not detrimental to follicular survival and growth of preantral and early antral follicles [35].

The reduced number of follicles ≥6 mm in T females suggests the final maturation of the preovulatory follicle is impaired. Given that *i)* the suppression was evident in number of follicles ≥6 mm, but not in the number up to 4 mm, and *ii)* in sheep, follicles up to 4 mm size are considered FSH dependent and those beyond 4 mm as LH dependent [28], the shift from FSH to LH dependency appears to be impaired. One possibility is that this reduction in preovulatory sized follicles might be a function of advancement in LH dependency and hence a requirement for LH. Because LH was not co-administered with FSH, the final transition to preovulatory size might be compromised. This premise is supported by the lack of reduction in preovulatory sized follicles in the T30-90 females [32], when follicles were stimulated concomitantly with LH and FSH. Findings from both studies (this study and Steckler et al. [32]) suggest the compromised preovulatory follicular development might be rescued with exogenous LH supplementation. It is unclear if this impairment is a function of reduced number of LH receptors or altered LH signaling, both are aspects yet to be studied in this model but implicated in women with PCOS [36–38].

Importantly, the fact that postnatal androgen antagonist, not insulin sensitizer, treatment was able to rescue the number of preovulatory-sized follicles supports the notion that *i)* compromised androgen receptor expression/function in growing follicles or surrounding ovarian stroma is detrimental to progression beyond a critical size (4 mm in this case) and *ii)* that blockade of androgen action with an androgen antagonist would help overcome follicular growth arrest. In previous studies, we found prenatal T treatment from days 30-90 of gestation increases granulosa cell androgen receptor expression in antral follicles and is supportive of functional ovarian hyperandrogenism [15]. Although insulin plays a role in follicular development [39–41], failure of insulin sensitizer treatment to rescue this follicular growth defect suggests this dysfunction is driven primarily by the androgen signaling imbalance within the growing follicle.

### Prenatal T programming of follicular steroid milieu

The opposing follicular size-related changes in intrafollicular T and estrogens in control females were similar to previous findings [20, 42]. A higher androgenic environment prevails in small follicles, with a shift towards a highly estrogenic milieu in preovulatory follicles; this is consistent with increased aromatase activity as follicles mature [42]. The transition from low E_2_ in smaller follicles to high E_2_ in larger follicles was the most striking change (4-fold increase) in the control group. Conversely, the E_2_ increase was of much lower magnitude (< 1-fold) in the T group. Because androgens are the main substrate for estrogen production, the reduced magnitude of E_2_ increase in T females may be driven by a reduced androgenic environment (T and A4) at earlier follicular stages. Androgen and estrogen receptors [17] and steroidogenic enzymes [43] are dysregulated in granulosa and theca cells of T30-90 sheep, and these disruptions remain to be determined in T60-90 females. It is also unclear whether the lower magnitude of E_2_ increase in T females contributes to the delayed onset of LH surge that was reported earlier in these females [29].

Prenatal T-induced disruptions in intrafollicular steroid milieu were also reported in non-human primates [11]. Controlled ovarian stimulation studies in rhesus monkeys found 15-35 days of T treatment starting on gestational days 40-44, but not days 100-115, reduced intrafollicular A4 and E_2_ concentrations in preovulatory sized follicles [11]. Although disruptions in intrafollicular A4 and E_2_ parallel findings from the current study, timing and duration of T exposure differ between the sheep and monkey study. Our earlier findings of reduced granulosa cell CYP19A1 expression in antral follicles in the T30-90 model [43] agrees with the reduced intrafollicular E_2_ in large antral follicles evidenced in the present study [43]. Considering the disrupted intrafollicular steroidal milieu of prenatal T-treated monkeys was accompanied by a reduction in oocyte competence [11], a similar intrafollicular disruption was evidenced in prenatal T-treated sheep is likely to be associated with compromised oocyte health. This, in fact, may explain the reduced fecundity in T60-90 females [19]. The strength of the present study is that the intrafollicular steroid milieu was identified in different size follicular classes as opposed to only the preovulatory follicular size in monkeys. Another strength is the parallel assessment of follicular growth at different time points during the ovarian stimulation protocol, which was helpful in dissecting out regulation of follicular growth from steroidogenesis.

Relative to interventions, postnatal flutamide treatment rescued follicular growth to where preovulatory follicle size was achieved, but treatment failed to ameliorate the disruptions in intrafollicular steroidal milieu. The steroidal transition from low E_2_ to a high E_2_ milieu between 3 to 6 mm size antral follicles seen in controls was not evident in T + F females. In addition, the directionality of change in intrafollicular concentrations of estrone and A4 in T + F animals, namely a reduction in both steroids in the 6 mm compared to 3 mm follicles as opposed to the increase in both steroids in the controls, point to an intrafollicular steroidal disruption that persists through antral follicle growth. The increase of T in the smaller 3 mm follicles of the T + F females may be a compensatory response to the blockade of androgen receptor signaling by flutamide treatment, which was present throughout the course of the study. Such a response would be analogous to the masculinizing effects of flutamide seen relative to other variables [44, 45]. To what extent the intrafollicular steroidal disruptions play a role in oocyte health and ultimately fertility remains unclear. Paradoxically, while flutamide treatment failed to ameliorate intrafollicular steroidal defects, the same treatment prevented pubertal advancement and enhanced preovulatory LH surge amplitude in the T30-90 females [24]. Considering T is an aromatizable androgen, the differing effects of flutamide in rescuing the various physiologic functions may be a function of whether androgen or estrogen (via aromatization) is the programming agent [31].

Despite the fact that T60-90 females are insulin resistant [13], the lack of rescue in the number of follicles that achieved a preovulatory size by rosiglitazone suggests the insulin pathway is not involved in growth of preovulatory follicles. In contrast, the intrafollicular steroid milieu of T + R follicles was more similar to that of the controls and is supportive of a role for insulin coupled with FSH as follicles mature in maintaining intrafollicular steroid balance [46]. The beneficial effects of insulin sensitizer therapies in enhancing insulin sensitivity and improving ovulatory function in women with PCOS [47, 48] may relate to normalization of intrafollicular steroidal milieu, as evidenced in the T + R animals.

In interpreting the impact of the interventions, it is important to recognize that GnRH antagonist treatment given prior to FSH stimulation to achieve a homogeneous follicular pool before FSH stimulation (as achieved in this study, Fig. 1) might have played a role in determining the impact of androgen antagonist and insulin sensitizer on follicular dynamics and the intra-follicular hormone milieu. However, considering that GnRH antagonist treatment is the same across treatments, any variability in the starting pool of follicles across treatment would suggest intrinsic ovarian differences originating from the T treatment and interventions respectively. It needs to be recognized that GnRH agonist and antagonist treatments are routinely used in standard IVF practices [49], and hence the approach taken with this study is consistent with this practice.

The outcomes achieved with the two interventions, namely the androgen antagonist helping rescue follicular growth and the insulin sensitizer partially rescuing intrafollicular steroidal milieu, suggest that both androgens and insulin may synergize in establishing optimal follicular growth and steroidogenesis. A combined intervention involving both may help compensate for any deficiency that one intervention has in order to achieve better success. While the finding in PCOS women is that combined treatment is more efficacious than monotherapies in treating anovulation [50] is supportive of this possibility, this remains to be tested.

## Conclusions

Prenatal T excess from days 60-90 of gestation impairs final follicular growth and intrafollicular milieu under controlled ovarian stimulation protocols. This indicates an inherent ovarian defect that may contribute to the lower fertility seen in these females [19]. The differential benefit of postnatal androgen antagonist and insulin sensitizer treatment in rescuing follicular growth and steroidogenesis, respectively, raises the possibility that combined therapies during adolescence and early adulthood may be beneficial in enhancing fertility, a premise that remains to be tested.

## References

[1] Womeshealth.gov. Polycystic ovary syndrome (PCOS) fact sheet. http://www.womenshealth.gov/publications/our-publications/fact-sheet/polycystic-ovary-syndrome.html Last updated: December 23, 2014. Last accessed: January 29, 2016.

[2] Rotterdam EA-SPCWG (2004). Revised 2003 consensus on diagnostic criteria and long-term health risks related to polycystic ovary syndrome. Fertil Steril.

[3] Zawadki J, Dunaif A. Diagnostic criteria for polycystic ovary syndrome: towards a rational approach. In: A D, JR G, FP H, GR M, eds. Polycystic ovary syndrome. Boston: Blackwell Scientific Publications; 1992. p. 377-84.

[4] Azziz R, Carmina E, Dewailly D, Diamanti-Kandarakis E, Escobar-Morreale HF, Futterweit W (2006). Positions statement: criteria for defining polycystic ovary syndrome as a predominantly hyperandrogenic syndrome: an Androgen Excess Society guideline. J Clin Endocrinol Metab.

[5] Michael AE, Glenn C, Wood PJ, Webb RJ, Pellatt L, Mason HD (2013). Ovarian 11beta-hydroxysteroid dehydrogenase (11betaHSD) activity is suppressed in women with anovulatory polycystic ovary syndrome (PCOS): apparent role for ovarian androgens. J Clin Endocrinol Metab.

[6] Barreca A, Del Monte P, Ponzani P, Artini PG, Genazzani AR, Minuto F (1996). Intrafollicular insulin-like growth factor-II levels in normally ovulating women and in patients with polycystic ovary syndrome. Fertil Steril.

[7] Lambert-Messerlian G, Taylor A, Leykin L, Isaacson K, Toth T, Chang Y (1997). Characterization of intrafollicular steroid hormones, inhibin, and follistatin in women with and without polycystic ovarian syndrome following gonadotropin hyperstimulation. Biol Reprod.

[8] Hossein G, Arabzadeh S, Hossein-Rashidi B, Hosseini MA (2012). Relations between steroids and AMH: impact of basal and intrafollicular steroids to AMH ratios on oocyte yield and maturation rate in women with or without polycystic ovary undergoing in vitro fertilization. Gynecol Endocrinol.

[9] Ambekar AS, Kelkar DS, Pinto SM, Sharma R, Hinduja I, Zaveri K (2015). Proteomics of follicular fluid from women with polycystic ovary syndrome suggests molecular defects in follicular development. J Clin Endocrinol Metab.

[10] Homburg R, Berkowitz D, Levy T, Feldberg D, Ashkenazi J, Ben-Rafael Z (1993). In vitro fertilization and embryo transfer for the treatment of infertility associated with polycystic ovary syndrome. Fertil Steril.

[11] Dumesic DA, Schramm RD, Peterson E, Paprocki AM, Zhou R, Abbott DH (2002). Impaired developmental competence of oocytes in adult prenatally androgenized female rhesus monkeys undergoing gonadotropin stimulation for in vitro fertilization. J Clin Endocrinol Metab.

[12] Siristatidis C, Sergentanis TN, Vogiatzi P, Kanavidis P, Chrelias C, Papantoniou N (2015). In Vitro Maturation in Women with vs. without Polycystic Ovarian Syndrome: A Systematic Review and Meta-Analysis. PLoS One.

[13] Padmanabhan V, Veiga-Lopez A (2013). Animal models of the polycystic ovary syndrome phenotype. Steroids.

[14] Abbott DH, Dumesic DA, Levine JE, Dunaif A, Padmanabhan V, Azziz JE, Nestler JE, Dewailly D (2006). Animal models and fetal programming of PCOS. Contemporary endocrinology: androgen excess disorders in women: polycystic ovary syndrome and other disorders.

[15] Ortega HH, Salvetti NR, Padmanabhan V (2009). Developmental programming: prenatal androgen excess disrupts ovarian steroid receptor balance. Reproduction.

[16] Salvetti NR, Ortega HH, Veiga-Lopez A, Padmanabhan V (2012). Developmental programming: impact of prenatal testosterone excess on ovarian cell proliferation and apoptotic factors in sheep. Biol Reprod.

[17] Ortega HH, Rey F, Velazquez MM, Padmanabhan V (2010). Developmental programming: effect of prenatal steroid excess on intraovarian components of insulin signaling pathway and related proteins in sheep. Biol Reprod.

[18] Veiga-Lopez A, Ye W, Padmanabhan V (2012). Developmental programming: prenatal testosterone excess disrupts anti-Mullerian hormone expression in preantral and antral follicles. Fertil Steril.

[19] Steckler TL, Roberts EK, Doop DD, Lee TM, Padmanabhan V (2007). Developmental programming in sheep: administration of testosterone during 60-90 days of pregnancy reduces breeding success and pregnancy outcome. Theriogenology.

[20] Veiga-Lopez A, Dominguez V, Souza CJ, Garcia-Garcia RM, Ariznavarreta C, Tresguerres JA (2008). Features of follicle-stimulating hormone-stimulated follicles in a sheep model: keys to elucidate embryo failure in assisted reproductive technique cycles. Fertil Steril.

[21] Domecq JP, Prutsky G, Mullan RJ, Sundaresh V, Wang AT, Erwin PJ (2013). Adverse effects of the common treatments for polycystic ovary syndrome: a systematic review and meta-analysis. J Clin Endocrinol Metab.

[22] Veiga-Lopez A, Lee JS, Padmanabhan V (2010). Developmental programming: insulin sensitizer treatment improves reproductive function in prenatal testosterone-treated female sheep. Endocrinology.

[23] Manikkam M, Crespi EJ, Doop DD, Herkimer C, Lee JS, Yu S (2004). Fetal programming: prenatal testosterone excess leads to fetal growth retardation and postnatal catch-up growth in sheep. Endocrinology.

[24] Padmanabhan V, Veiga-Lopez A, Herkimer C, Abi Salloum B, Moeller J, Beckett E (2015). Developmental programming: prenatal and postnatal androgen antagonist and insulin sensitizer interventions prevent advancement of puberty and improve LH surge dynamics in prenatal testosterone-treated sheep. Endocrinology.

[25] Veiga-Lopez A, Gonzalez-Bulnes A, Garcia-Garcia RM, Dominguez V, Cocero MJ (2005). The effects of previous ovarian status on ovulation rate and early embryo development in response to superovulatory FSH treatments in sheep. Theriogenology.

[26] Veiga-Lopez A, Wurst AK, Steckler TL, Ye W, Padmanabhan V (2014). Developmental programming: postnatal estradiol amplifies ovarian follicular defects induced by fetal exposure to excess testosterone and dihydrotestosterone in sheep. Reprod Sci.

[27] Jackson LM, Mytinger A, Roberts EK, Lee TM, Foster DL, Padmanabhan V (2013). Developmental programming: postnatal steroids complete prenatal steroid actions to differentially organize the GnRH surge mechanism and reproductive behavior in female sheep. Endocrinology.

[28] van den Hurk R, Zhao J (2005). Formation of mammalian oocytes and their growth, differentiation and maturation within ovarian follicles. Theriogenology.

[29] Sharma TP, Herkimer C, West C, Ye W, Birch R, Robinson JE (2002). Fetal programming: prenatal androgen disrupts positive feedback actions of estradiol but does not affect timing of puberty in female sheep. Biol Reprod.

[30] Savabieasfahani M, Lee JS, Herkimer C, Sharma TP, Foster DL, Padmanabhan V (2005). Fetal programming: testosterone exposure of the female sheep during midgestation disrupts the dynamics of its adult gonadotropin secretion during the periovulatory period. Biol Reprod.

[31] Padmanabhan V, Veiga-Lopez A (2011). 2011 Developmental origin of reproductive and metabolic dysfunctions: androgenic versus estrogenic reprogramming. Semin Reprod Med.

[32] Steckler TL, Lee JS, Ye W, Inskeep EK, Padmanabhan V (2008). Developmental programming: exogenous gonadotropin treatment rescues ovulatory function but does not completely normalize ovarian function in sheep treated prenatally with testosterone. Biol Reprod.

[33] Gonzalez-Bulnes A, Santiago-Moreno J, Cocero MJ, Lopez-Sebastian A (2000). Effects of FSH commercial preparation and follicular status on follicular growth and superovulatory response in Spanish Merino ewes. Theriogenology.

[34] Gervásio CG, Bernuci MP, Silva-de-Sá MF, Rosa-E-Silva AC. The role of androgen hormones in early follicular development. ISRN Obstet Gynecol. 2014;2014:818010. doi:10.1155/2014/818010. 10.1155/2014/818010 PMC4003798 25006485

[35] Vendola KA, Zhou J, Adesanya OO, Weil SJ, Bondy CA (1998). Androgens stimulate early stages of follicular growth in the primate ovary. J Clin Invest.

[36] Liu N, Ma Y, Wang S, Zhang X, Zhang Q, Zhang X (2012). Association of the genetic variants of luteinizing hormone, luteinizing hormone receptor and polycystic ovary syndrome. Reprod Biol Endocrinol.

[37] Comim FV, Teerds K, Hardy K, Franks S (2013). Increased protein expression of LHCG receptor and 17α-hydroxylase/17-20-lyase in human polycystic ovaries. Hum Reprod.

[38] McAllister JM, Modi B, Miller BA, Biegler J, Bruggeman R, Legro RS (2014). Overexpression of a DENND1A isoform produces a polycystic ovary syndrome theca phenotype. Proc Natl Acad Sci USA.

[39] Poretsky L, Bhargava G, Kalin MF, Wolf SA (1988). Regulation of insulin receptors in the human ovary: in vitro studies. J Clin Endocrinol Metab.

[40] Seto-Young D, Avtanski D, Strizhevsky M, Parikh G, Patel P, Kaplun J (2007). Interactions among peroxisome proliferator activated receptor-gamma, insulin signaling pathways, and steroidogenic acute regulatory protein in human ovarian cells. J Clin Endocrinol Metab.

[41] Kayampilly PP, Menon KM (2007). Follicle-stimulating hormone increases tuberin phosphorylation and mammalian target of rapamycin signaling through an extracellular signal-regulated kinase-dependent pathway in rat granulosa cells. Endocrinology.

[42] Tsonis CG, Carson RS, Findlay JK (1984). Relationships between aromatase activity, follicular fluid oestradiol-17 beta and testosterone concentrations, and diameter and atresia of individual ovine follicles. J Reprod Fertil.

[43] Padmanabhan V, Salvetti NR, Matiller V, Ortega HH (2014). Developmental programming: prenatal steroid excess disrupts key members of intraovarian steroidogenic pathway in sheep. Endocrinology.

[44] Mylchreest E, Sar M, Wallace DG, Foster PM (2002). Fetal testosterone insufficiency and abnormal proliferation of Leydig cells and gonocytes in rats exposed to di(n-butyl) phthalate. Reprod Toxicol.

[45] Herman RA, Measday MA, Wallen K (2003). Sex differences in interest in infants in juvenile rhesus monkeys: relationship to prenatal androgen. Horm Behav.

[46] Chaves RN, Duarte AB, Rodrigues GQ, Celestino JJ, Silva GM, Lopes CA (2012). The effects of insulin and follicle-simulating hormone (FSH) during in vitro development of ovarian goat preantral follicles and the relative mRNA expression for insulin and FSH receptors and cytochrome P450 aromatase in cultured follicles. Biol Reprod.

[47] Pasquali R, Gambineri A (2013). Insulin sensitizers in polycystic ovary syndrome. Front Horm Res.

[48] Naderpoor N, Shorakae S, de Courten B, Misso ML, Moran LJ, Teede HJ (2015). Metformin and lifestyle modification in polycystic ovary syndrome: systematic review and meta-analysis. Hum Reprod Update.

[49] Garcia-Velasco JA, Fatemi HM (2015). To pill or not to pill in GnRH antagonist cycles: that is the question!. Reprod Biomed Online.

[50] Ibáñez L, de Zegher F (2006). Low-dose flutamide-metformin therapy for hyperinsulinemic hyperandrogenism in nonobese adolescents and women. Fertil Steril.

